# COVID-19 pandemic, Uganda’s story

**DOI:** 10.11604/pamj.supp.2020.35.2.23433

**Published:** 2020-05-27

**Authors:** David Lagoro Kitara, Eric Nzirakaindi Ikoona

**Affiliations:** 1 Harvard University, Harvard TH Chan School of Public health, Department of Global Health, USA; 2 Gulu University, Faculty of Medicine, Department of Surgery, Uganda; 3 African Field Epidemiology Network

**Keywords:** COVID-19, Uganda, response, leadership, multiple public health interventions

## Abstract

As the COVID-19 pandemic continues to ravage health care systems, economies, livelihoods, and cultures across the world, responses across countries have varied greatly. Uganda adopted its own model taking into consideration its culture, values, environment, socio-economic activities, beliefs, previous successful epidemic experience, and appears a hybrid policy to the Norwegian model. This model of response is perhaps based on Uganda’s long experience in successful control of many previous epidemics which afflicted it and the neighboring countries, e.g, HIV and AIDs in the 1980s, Measles in the 1990s, Hepatitis B in the 2000s, Ebola in 2000, 2017 and 2018 and Marburg in 2018. In our view the near complete lockdown through shutting down air, road, water travels and congregate settings as well as the restriction of people’s movement through the stay home policy may have, so far, played a significant role in this pandemic containment and control. Most notable is that there is an established and clear leadership structure, experienced health workforce, good political will, enabling environment, and good epidemic response by the population. Even though one can reasonably argue that the numbers of COVID-19 cases seen in Uganda so far, are not anywhere close to those large numbers seen in the USA, Asia and other European countries, Uganda’s story on how it is managing the pandemic is worth sharing as it might provide useful lessons for future public health interventions to a pandemic of this magnitude, particularly in low-resource settings. Uganda’s President continued to provide national leadership, guidance, and coordination to the COVID-19 National task force for the response. The President and Ministry of Health authorities employed both electronic and social media such as radios, music, Televisions, SMS messages, twitters, group emails, and WhatsApp messages to engage, mobilize, and sensitize the population on COVID-19 preventive interventions through provision of regular updates. In conclusion, simultaneous multiple public health interventions through a structured leadership may in part contribute to reasonable and timely control of a pandemic such as COVID-19.

## Commentary

As the COVID-19 pandemic continues to ravage health care systems, economies, livelihoods, and cultures across the world, responses across countries have varied greatly. Of notable interest is the two approaches to control the spread of SARS-CoV-2, the virus that causes COVID-19, implemented by the two Scandinavian countries; no lockdown “Swedish model” and lockdown “Norwegian model”. As of April 30, 2020, Sweden had conducted 120,000 tests, confirmed 23,216 COVID-19 cases (prevalence 19.35%), and registered 2,854 deaths (CMR 12.29%) with 11.8 tests/1,000 conducted as compared to Norway with 172,586 tests, confirmed 7,955 COVID-19 cases (prevalence 4.61%), and registered 215 deaths (CMR 2.70%) with 31.2 tests/1,000 conducted respectively [1]. Perhaps premised on its culture, values and community trust, the Swedish Model is different from the Norwegian approach which is anchored on preventing the virus from infecting the population as other interventions await to be developed, or the virus becomes self-limited, and so, implemented the lockdown policy. The marked differences in the success to contain the 1918 influenza pandemic between communities of Philadelphia and St. Louis in USA is one good example used commonly in public health discussions [2]. It highlights the importance of timely response and simultaneous use of multiple public health interventions in successful control of epidemics [2]. Furthermore, it is noted that the impact of an epidemic is largely determined by the number of persons infected, the transmissibility of the infection and the spectrum of clinical severity [2,3]. The approach adopted by each country should be based on epidemiological principles of flattening the curve in an epidemic outbreak, ensuring that the prevalence of the disease does not overwhelm the health care systems to care and manage all patients with minimal deaths [2].

Notably, Uganda adopted its own model taking into consideration its culture, values, environment, socio-economic activities, beliefs, previous successful epidemic experience, and appears a hybrid policy to the Norwegian model. Even though one can reasonably argue that the numbers of COVID-19 cases seen in Uganda so far, are not anywhere close to those large numbers seen in the USA, Asia and other European countries, Uganda´s story on how it is managing the pandemic is worth sharing as it might provide useful lessons for future public health interventions to a pandemic of this magnitude, particularly in low-resource settings. Following WHO declaration of COVID-19, a pandemic on March 11, 2020, there was reportedly massive influx of returnees into Uganda, which forced the government through its Ministry of Health (MoH) to swiftly institute enhanced surveillance through mandatory screening for flu-like symptoms and taking of temperature on travelers at its airports to prevent the importation of cases into the country. The Ugandan government also implemented a 14-day mandatory institutional quarantine and testing for COVID-19 on day 14 for all travelers from high-risk countries, also referred to as category 1 and 2 countries depending on the number of cases they had at the time [1]. The government also traced all persons who had come from high-risk countries early on for a mandatory 14-day quarantine and appropriate testing. Those who did not complete their mandatory 14-day quarantine were traced to the communities, returned for quarantine and re-tested. Those who tested negative continued to be monitored by the MoH authorities using mobile phones. In addition, community contacts of returnees from high-risk countries were also line-listed and monitored by the MoH authorities.

Within eleven days, on March 22, 2020, the Ministry of health authorities had instituted multiple other public health interventions, including closing all international borders and restricting population movement through a well-enforced stay home policy. Throughout March and April, country-wide contact tracing and testing for COVID-19 continued to be conducted at a centralized laboratory located at Uganda Virus Research Institute (UVRI) in Entebbe. Congregate settings such as schools, recreation centres, sports, parks, and places of worship, bars, markets, hotels, public transport, and other public gatherings which attract large numbers of people were closed, and the government imposed a curfew beginning 7:00 PM to 6:00 AM EST for the population. Uganda’s President continued to provide national leadership, guidance, and coordination to the COVID-19 National task force for the response. The President and Ministry of Health authorities employed both electronic and social media such as radios, music, Televisions, SMS messages, twitters, group emails, and WhatsApp messages to engage, mobilize, and sensitize the population on COVID-19 preventive interventions through provision of regular updates. Lower down, COVID-19 committees at the district-level were formed and supported by the National task force to implement the response interventions within their communities. Although treatment of confirmed cases were initially provided only in two national-level health facilities, as time went on, it got decentralized to regional centres to enhance efficiency and equity.

**Figure 1:**
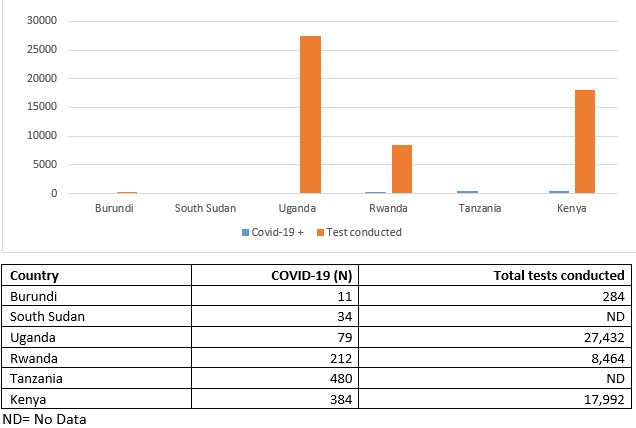
Shows the prevalence of COVID-19 among the East African countries. The numbers are generally low and Uganda has conducted more tests than all the other countries and implemented a complete lockdown policy

At 79 COVID-19 cases from March to date, Uganda has relatively fewer cases compared to Kenya (384), Tanzania (480), and Rwanda (212) (Figure 1). The current situation of Uganda having relatively fewer cases compared to its neighbors is commendable and can generate some important lessons for the region. In our view the near complete lockdown through shutting down air, road, water travels and congregate settings as well as the restriction of people´s movement through the stay home policy may have, so far, played a significant role in this pandemic containment and control. We also posit that Uganda´s early successes in controlling the pandemic may be attributable to the strong leadership from the Ugandan President and Ministry of Health, who took up the leadership to coordinate the COVID-19 national response. This model of response is perhaps based on Uganda’s long experience in successful control of many previous epidemics which afflicted it and the neighboring countries, e.g, HIV and AIDs in the 1980s, Measles in the 1990s, Hepatitis B in the 2000s, Ebola in 2000, 2017, and 2018 and Marburg in 2018. Most notable is that there is an established and clear leadership structure, experienced health workforce, good political will, enabling environment, and good epidemic response by the population.

To date, Uganda has 79(0.28%) confirmed COVID-19 cases, conducted 28,000 tests with 47(59.5%) recoveries, registered 0 deaths (CMR 0.0%), and the country is looking forward to re-opening its economy in a phased manner. According to Ugandan Ministry of Health official statistics, 27(34.2%) of confirmed COVID-19 cases in Uganda were from international truck drivers from neighbouring Countries. It is reported that because of regional agreements, international truck drivers were not required to quarantine, and this presents a significant challenge to COVID-19 prevention and control not only for Uganda but for the region as a whole. Finally, Uganda is currently conducting Household studies to help it make an informed decision on how to ease or extend the lockdown it had imposed on residents since March 22, 2020. These studies are expected to help define the role that subclinical, asymptomatic, and mild infections play in transmission to inform evidence-based decisions about prioritizing the control measures [3]. These measures that depend on identification and isolation of symptomatic persons will continue at border points, health facilities, communities, and airports because these persons have the primary role in the transmission of this infection [3,4].

## Conclusion

Simultaneous multiple public health interventions through a structured leadership may in part contribute to reasonable and timely control of a pandemic such as COVID-19.

**Recommendation:** these authors however wish to note that well aware of all these achievements in the control of COVID-19, Uganda being a low-income country with high burden of diseases, it shouldn´t take its eyes-off the other epidemic proned diseases which afflict its population; Malaria, TB, HIV and AIDS, diarrhoeal diseases and diseases of life-style such as Hypertension and Diabetes Mellitus.

## Competing interests

The author declares no competing interests.
